# Primary Malignant Melanoma of the Esophagus in a 76-Year-Old Female

**DOI:** 10.7759/cureus.23250

**Published:** 2022-03-17

**Authors:** Simon Kashfi, Naimisha Marneni, Shorabh Sharma, Ivette Vigoda

**Affiliations:** 1 Internal Medicine, City University of New York (CUNY) School of Medicine, New York, USA; 2 Internal Medicine, St. Barnabas Hospital Health System, Bronx, USA; 3 Oncology, St. Barnabas Hospital Health System, Bronx, USA

**Keywords:** primary malignant melanoma, dysphagia, nivolumab, melanoma, esophageal melanoma

## Abstract

Mucosal melanomas represent about 1% of all melanoma cases. Primary malignant melanoma of the esophagus (PMME) is a rare and deadly condition, with only about 339 cases reported in the literature. Esophageal melanoma usually presents with progressively worsening dysphagia, and patients often present late in the disease course. Esophageal melanoma can be treated with surgical resection, chemotherapy, targeted therapy, or immunotherapy depending on the stage and tumor mutations. However, due to the rarity of the disease, no trials have been performed to deliver a gold standard of treatment. We present the case of a 76-year-old female who was diagnosed with metastatic primary malignant melanoma of the esophagus and underwent treatment with nivolumab, a PD-1 receptor antagonist.

## Introduction

Melanomas are malignant tumors arising from pigment cells called melanocytes that most often occur on the skin [1]. The incidence of malignant melanoma was 20.1/100,000 in 2007 [2]. It occurs mostly in light-skinned populations due to differences in skin pigmentation. The most common locations for cutaneous malignant melanoma are sex-dependent. Men tend to have lesions on their backs, while women on their arms and legs [2]. The most important risk factor for malignant melanoma is exposure to UV-B radiation [2,3]. Other risk factors include increased number and size of melanocytic nevi, sunburns suffered early in life, and classic phenotypic characteristics such as freckles and fair hair, skin, and eyes [2,3]. Additionally, melanomas can rarely occur at mucosal surfaces, representing 1% of melanoma cases [4]. Mucosal melanomas can occur because melanocytes are derived from neural crest cells and migrate to various sites [1]. Mucosal melanomas have been reported to occur in the gastrointestinal tract, respiratory tract, male and female genitourinary tracts, and mouth. Head and neck mucosal melanomas are the most common. The literature surrounding mucosal melanomas is primarily composed of case reports and case series due to the rarity of the condition [1]. Primary malignant melanoma of the esophagus (PMME) accounts for 0.1%-0.2% of primary esophageal neoplasms and is associated with poor prognosis [5]. By 2016, there had only been 339 cases reported in the literature [6]. We present the case of a 76-year-old female who was diagnosed with PMME after presenting to the emergency department with several months of dysphagia.

## Case presentation

The patient is a 76-year-old female nonsmoker with no reported medical history who presented to our hospital with six months of chronic, worsening, nonradiating epigastric pain that was 10/10 in severity that was not associated with food intake. The symptoms initially began as difficulty with swallowing liquids and early satiety. They progressed to difficulty with solids and eventually to sialorrhea, as she was unable to swallow and had to spit liquids out. During this period, she experienced nearly 40-pound weight loss with associated anorexia and fatigue. On our initial clinical interaction, she was noted to be cachectic, expectorating constantly, but in no acute distress. There was no palpable lymphadenopathy.

**Figure 1:**
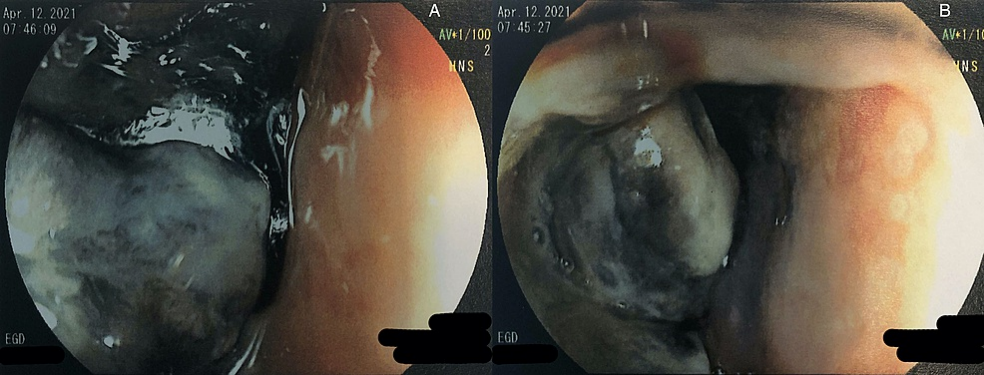
A and B: Visualization of the pigmented esophageal mass during EGD

**Figure 2:**
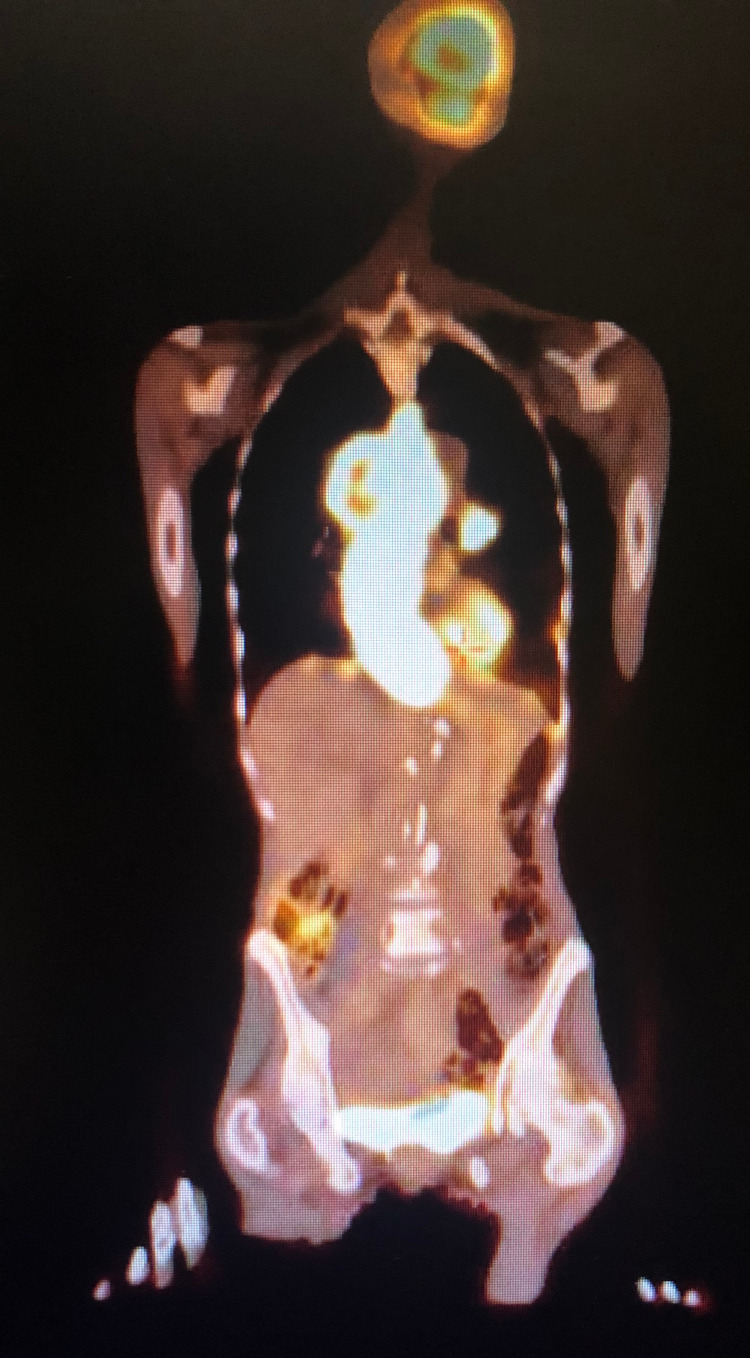
PET scan with significant involvement of the esophagus

Her initial laboratory results were remarkable for normocytic anemia secondary to anemia of chronic disease with components of iron deficiency and marked hypoalbuminemia. Hemoglobin was 10.3 g/dL, and albumin was 2.6 g/dL. CT scan of the abdomen was significant for an intraluminal esophageal mass measuring 4.9 × 4.2 cm axially and 12 cm in the craniocaudal dimension, originating in the subcarinal region and extending to the gastroesophageal junction. The mass compressed but did not occlude the right atrium and lower pulmonary veins. The patient underwent an esophagogastroduodenoscopy (EGD) for further evaluation, which showed a pigmented and lobulated esophageal mass starting at 21 cm from the incisors, with the endoscope unable to pass beyond 40 cm from incisors with the esophageal mass still being visualized (Figure 1). The biopsy samples taken were consistent with malignant melanoma supported by positive staining with SOX10, HMB-45, Melan A, and vimentin immunohistochemical markers, along with Fontana Masson special stain. Tumor cells were negative for p63, p40, CK7, and CK20 immunostains and negative for iron and mucin special stains. BRAF V600 mutation was not detected. Additionally, KIT activating mutation, NRAS mutation, and NTRK fusion mutation were not detected. PET scan revealed a metastatic melanoma predominantly involving the esophagus, mediastinal lymph nodes, and bony disease consistent with stage IV disease (Figure 2). There was no evidence of brain metastasis.

After evaluation by a multidisciplinary team, a combined decision was made by the family to initiate palliative chemoradiation. Subsequently, the patient underwent placement of a gastrostomy tube to maximize the quality of life given dysphagia with severe malnutrition and anticipated radiation, which could likely worsen the dysphagia. After discussion at an interdisciplinary melanoma tumor board, the patient was started on single-agent nivolumab given an Eastern Cooperative Oncology Group performance status score of 4. She received two doses of nivolumab 480 mg, four weeks apart, with no improvement of symptoms, and continued decondition, suggesting progressive disease not responding to treatment. The patient never received radiation because she missed her appointment with the radiation oncologist. Thereafter, an informed decision was made by the family to transition to hospice care. Death occurred two months after the last dose of nivolumab. The time from initial presentation to initiation of treatment was about one month, and the death occurred about 4.5 months after diagnosis.

## Discussion

The most common presenting symptom in PMME, as noted in the existing literature and shown in several case reports, is dysphagia [1,5-9]. This patient presented with several months of dysphagia, similar to the series of patients described by Wang et al. [6]. Patients often present only when their dysphagia has gotten too severe for them to manage, and unfortunately, this is often too late. It was at this point in our case that the patient presented. She received a CT scan and subsequent EGD, which was to visualize the mass found on CT and take a biopsy sample.

EGD can reveal a pigmented mass, such as our patient had [8]. An ulcerated mass is a predictor of poor prognosis [7]. However, there are cases where the mass is not pigmented and is still found to be melanoma [5,9-11]. In these cases, the diagnosis of primary malignant amelanotic melanoma of the esophagus is made when there are no melanocytes on microscopic examination [11]. Our patient’s mass was located starting 21 cm from the incisors and was still able to be visualized at 40 cm from the incisors, at which point the tube was no longer passed. This location is classic for PMME, which occurs most often at the distal third of the esophagus [1,5,7,8,11].

Histologically, the diagnosis of melanoma was supported by positive staining with SOX10, HMB-45, Melan A, and vimentin immunostains and positive staining with Fontana Masson special stain. HMB-45 is an indicator of active melanosome formation [11] and, with Melan A, provides good specificity [8]. Classically, cutaneous melanomas are positive for mutations in BRAF and NRAS [3]. Specifically, the BRAF V600E mutation is responsible for up to 90% of BRAF mutations [12]. However, the rate of BRAF mutations is much less common in mucosal melanomas; white KIT mutations are more common [3]. BRAF mutations occur in 5%-20% of PMME. BRAF is an oncogene in the MAP kinase pathway, which is the downstream signaling pathway for various growth factors. In a study of 76 patients with PMME, nine were positive for C-KIT mutation, five for BRAF, and five for NRAS [6]. Our patient’s sample was negative for the BRAF V600E mutation, KIT activating mutation, NRAS mutation, and NTRK fusion. This is important because it did not offer any targetable mutations [13].

The staging of cancer helps guide its treatment. PMME is treated with surgical resection if the disease is at a limited stage [6,8,10], and some patients have had good outcomes [5]. However, even in those who had an excision considered to be complete, 89.7% had recurrences, with a median recurrence time of 4.5 months [6]. For metastatic disease, there is no preferred pharmacological regimen. Wang et al. reviewed 60 patients with PMME who were treated with either chemotherapy, targeted therapy, or immunotherapy. The chemotherapy group received either dacarbazine, temozolomide, or paclitaxel + carboplatin. The targeted therapy group received imatinib. The immunotherapy group received PD-1 checkpoint inhibitors. Of the patients in the immunotherapy group, 75% had a partial response to treatment, with a median response duration of 11.4 months [6]. Our patient received nivolumab, a PD-1 receptor antagonist. Unfortunately, her disease progressed, and treatment was stopped after two months, with death soon after.

## Conclusions

Primary malignant melanoma of the esophagus is a rare form of esophageal cancer. Prognosis is poor and depends, among others, on the stage at diagnosis. There is a high risk of recurrence even in those who had a primary surgical resection. Those with metastatic disease and good performance status are candidates for immunotherapy with PD-1 checkpoint inhibitors, although the efficacy of these drugs is variable. Further research is warranted to determine optimal treatment protocols.
